# Overdiagnosis of vaccine allergy: Skin testing and challenge at a public specialized unit (CRIE) in Rio de Janeiro, Brazil

**DOI:** 10.1016/j.jacig.2023.100101

**Published:** 2023-03-24

**Authors:** Luciana Gomes Pedro Brandão, Mari Tuyama, Flávio de Carvalho, Ananza Taina da Silva Santos, Alberto dos Santos de Lemos, Marcellus Dias da Costa, Emersom Cicilini Mesquita, José Cerbino-Neto, Margareth Catoia Varela, Pedro Emmanuel Alvarenga Americano do Brasil, Angélica Varela Rondon

**Affiliations:** a Reference Center for Special Immunobiologicals, National Institute of Infectious Disease Evandro Chagas, Rio de Janeiro, Brazil; b Health Surveillance and Immunization Research Unit, National Institute of Infectious Disease Evandro Chagas, Rio de Janeiro, Brazil; c D’Or Institute for Research and Education, Rio de Janeiro, Brazil

**Keywords:** Adults, allergy, vaccine, immunization, hypersensitivity, skin allergic test, prick test, adverse event

## Abstract

**Background:**

Vaccination is an extremely safe public health intervention, but rare IgE-mediated adverse events must be identified to avoid the risk of anaphylaxis in the event of reexposure. However, using only clinical history to diagnose previous allergic reactions may lead to overdiagnosis of vaccine allergy and even to the use of medical exemptions as a subterfuge to mandatory vaccination.

**Methods:**

We conducted a retrospective study to describe the outcomes of patients with a history of vaccine or vaccine component allergy who were evaluated at our unit from 2011 to 2017. Data on allergy history, skin test results, vaccines prescribed, and adverse events were retrieved from the medical records at the Centro de Referência para Imunobiológicos Especiais (Reference Center of Special Immunobiologicals)–Fiocruz, in Rio de Janeiro, Brazil.

**Results:**

Of 34 adults with history of allergy to vaccine or vaccine components, 32 (94.1%) were successfully vaccinated without serious adverse events after our evaluation. In 12 patients (35%), the time elapsed between the allergy symptoms and evaluation in the Centro de Referência para Imunobiológicos Especiais–Fiocruz was more than 10 years.

**Conclusion:**

Specialized care and use of skin tests allowed safe vaccination of the majority of patients. An objective, systematic evaluation of a history of vaccine allergy can prevent its improper use to avoid mandatory vaccination and reduce missed opportunities for immunization.

Individuals with a history of potential IgE-mediated hypersensitivity reactions after immunization represent a public health problem, affecting individual and community health.^1^^,^^2^ Immediate-type hypersensitivity reactions are the result of release of mediators from mast cell granules (degranulation) into local tissues or the systemic circulation, which typically begin within minutes to an hour after vaccination but can, in rare instances, be delayed beyond this time frame. Most such reactions are IgE-mediated. Clinical protocols aimed at addressing those individuals have already been proposed, and when implemented, they tend to reduce the number of unvaccinated individuals.^1^^,^^3^^,^^4^ Allergy skin tests (prick tests and intradermal tests) with the vaccine or its components alone (eg, egg, gelatin, latex, and the fungus *Saccharomyces cervisiae*) are a central part of the clinical investigation and decision making, with no reports of vaccine anaphylaxis in patients with negative skin test results before the COVID-19 vaccine.^4^, ^5^, ^6^ Even with a positive test result, a graded dose protocol could be used after a thorough risk-benefit analysis has been conducted and patient consent has been obtained.^5^^,^^7^

In Brazil, individuals with potential IgE-mediated hypersensitivity reactions can be referred to public specialized units called Centro de Referência para Imunobiológicos Especiais (CRIE) units, which are a branch of the National Immunization Program, Ministry of Health. The CRIE units offer special vaccines for patients with chronic diseases and are the units responsible for evaluation of adverse events after immunization.^8^ A total of 52 CRIE units are available in the country, but the resources available in each unit are very heterogeneous. Most units have limited human resources, with no allergist on the team. Here we describe the protocol and outcomes of vaccine skin testing and challenge of adults with potential IgE-mediated hypersensitivity reactions who were referred to a CRIE unit from the National Institute of Infectious Disease Evandro Chagas–Fiocruz (INI), which was implemented in 2011 with a multidisciplinary team (infectious disease specialist and allergist).

## Methods

A retrospective review of all skin testing and/or challenge performed in adults aged 18 or years older who sought care at a CRIE unit from the INI in Rio de Janeiro, Brazil, over a 7-year period (from January 2011 to December 2017) is presented.

The study was approved by the Human Research Ethics Committee of the National Institute of Infectious Diseases, Rio de Janeiro, Brazil (approval no. 80901817.9.0000.5262).

### Study population

Subjects were identified through a database search for skin testing and/or challenge in the INI electronic medical records. Additional data, including sex, age, allergy-triggering vaccine or vaccine component, allergy symptoms, skin test results, outcome of vaccination, and time of vaccine delay due to allergy history, were collected by medical staff using Research Electronic Data Capture (REDcap), a mature and secure web platform for the construction and management of online surveys and databases.^9^ Notes were reviewed by trained data abstracters. Referral to a CRIE unit was at the discretion of the patient’s primary care physician and depended on the patient’s history of clinical reaction, coexisting comorbidities, and requirement for further vaccines. The decision to perform challenges with or without skin testing was shared between the infectious disease specialist and allergist and was based on the patient's history. When we could not rule out IgE-mediated reactions because of memory bias (because many reactions had occurred many years before), we preferred to proceed with allergy tests. Individuals were included in the study analysis if they were aged 18 years or older and had undergone either skin testing and/or challenge to either a suspect vaccine or vaccine with a suspect antigen.

### Skin testing

Skin prick testing (SPT) and/or intradermal testing (IDT) were performed at a CRIE unit on the same day by an allergist on the medical staff.^5^^,^^10^^,^^11^

Skin tests to evaluate immediate hypersensitivity were performed in 2 steps by using a diluted concentration (1:10) of the vaccine, lightly pricking the skin with a lancet, and administering a neat (undiluted [1:1]) concentration of the vaccine. The SPT result was read after 15 minutes between steps and considered positive when the wheal size was greater than 3 mm, with an appropriate negative control (saline solution) and positive control (histamine, 10 mg/mL).

If the SPT result was negative, IDT was performed with 0.02 mL of diluted (1:100) concentration of vaccine injected intradermally into the skin, with the result read after 20 minutes. A negative control was performed. The IDT result was considered positive when the final size of the papule was at least 2-fold the size of the initial papule, as recommended by the Brazilian Association of Allergy and Immunology.^12^ In patients with a positive SPT result, the IDT was not performed on that sample. Testing to excipient was not performed; however, an alternative vaccine (when available) was tested in those cases in which a positive skin test was found in the first instance.

### Challenge protocol

Patients were challenged with a single full vaccine dose. The challenge observation period was determined by the referring specialist, with a minimum of 30 minutes after the final dose.

## Results

**Fig 1:**
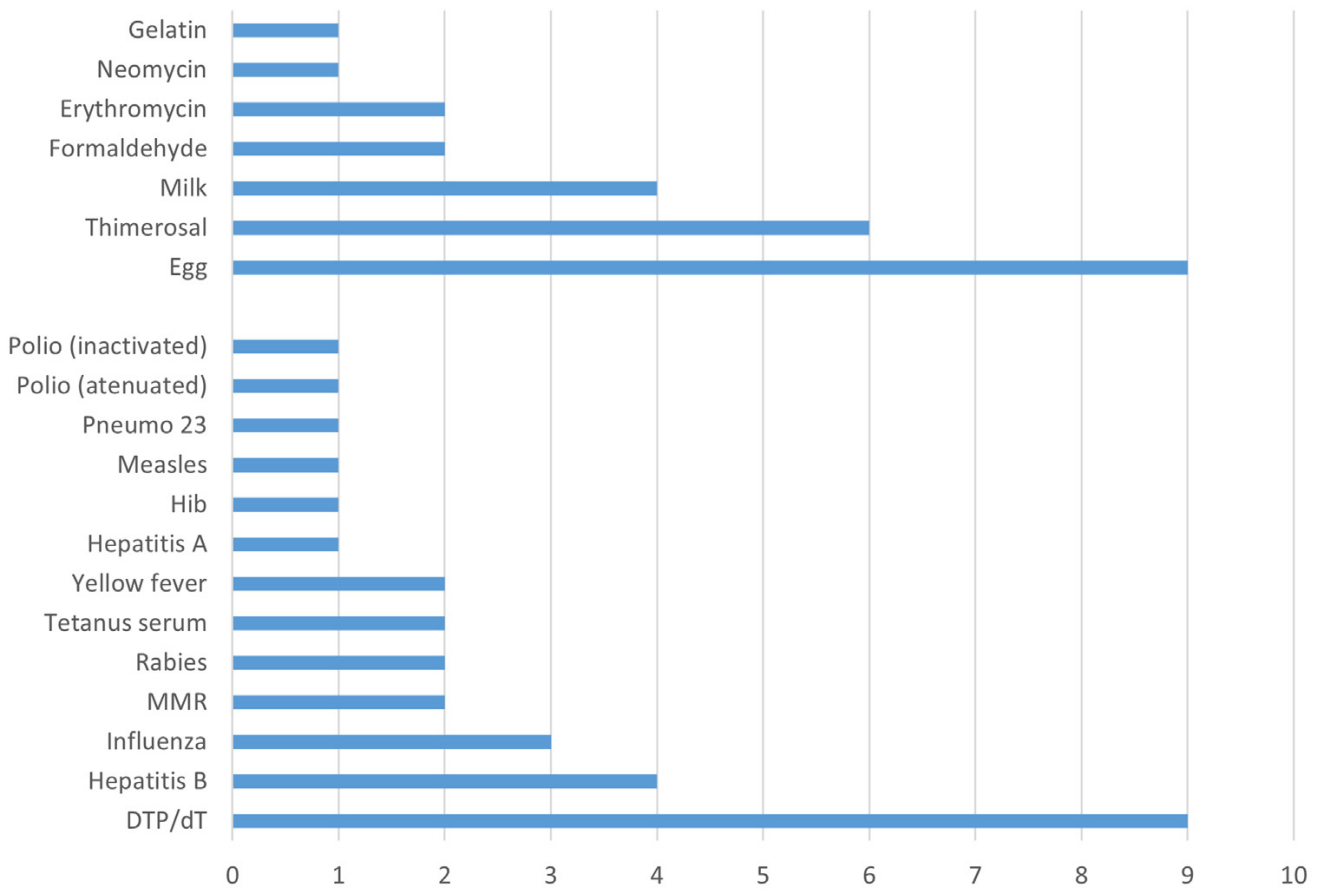
Number of allergy events in response to vaccine and/or vaccine components in 34 patients.

A total of 34 patients were included in this study; 19 (55.9%) of the 34 had a history of allergy to vaccine, 21 (61.8%) had a history of allergy to a vaccine component, and 6 (17.7%) had a history of allergy to both (Fig 1). Most of the patients were female (76.5%), and the median patient age was 40.1 years (SD = 15.53 years).

**Fig 2:**
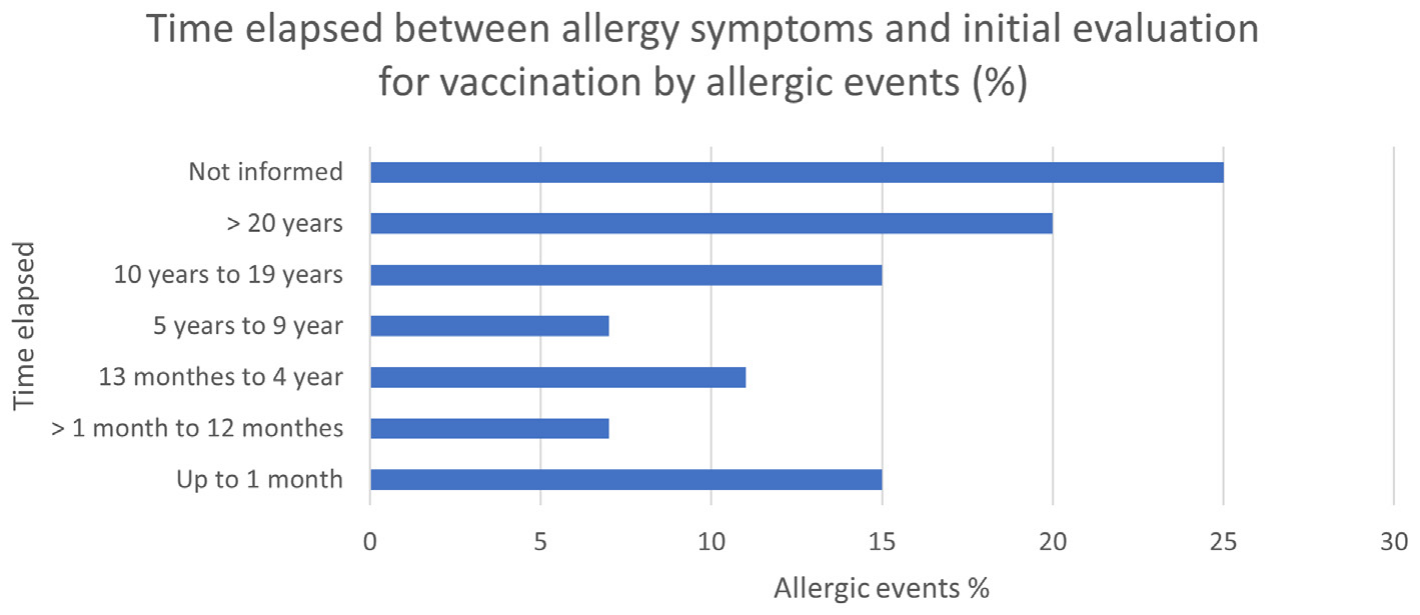
Time elapsed between allergy symptoms and initial evaluation for vaccination by allergic events (%).

There were 55 allergy events registered; 25 of them (45.5%) were classified as potentially IgE-mediated on the basis of time of onset (up to 6 hours after exposure) and/or symptom characteristics (urticarial rash, angioedema, and hypotension/shock). The most frequent vaccine and vaccine components associated with allergy were the diphtheria, tetanus and whole-cell pertussis (DTP) vaccine, adult diphtheria and tetanus (dT) vaccine, and egg, respectively (Fig 1), and the most frequent symptoms are described in Table I. In 35% of cases, the time elapsed between the patient's allergic reaction and first consultation in a CRIE unit for evaluation was more than 10 years, reflecting a delay in vaccine schedules (Fig 2).

**Table I:** Patient-informed allergy symptoms in response to vaccine or vaccine components

Symptom	Vaccine	Vaccine components
Dyspnea	0	3
Angioedema	11	2
Glottis edema	2	3
Application site reaction	3	3
Hypotension/vascular collapse	1	0
Itching	16	0
Undefined rash	6	7
Macular rash	0	6
Urticarial rash	7	0
Syncope/loss of consciousness	0	4
Positive allergic test result	3	0
Others	4	6

**Fig 3:**
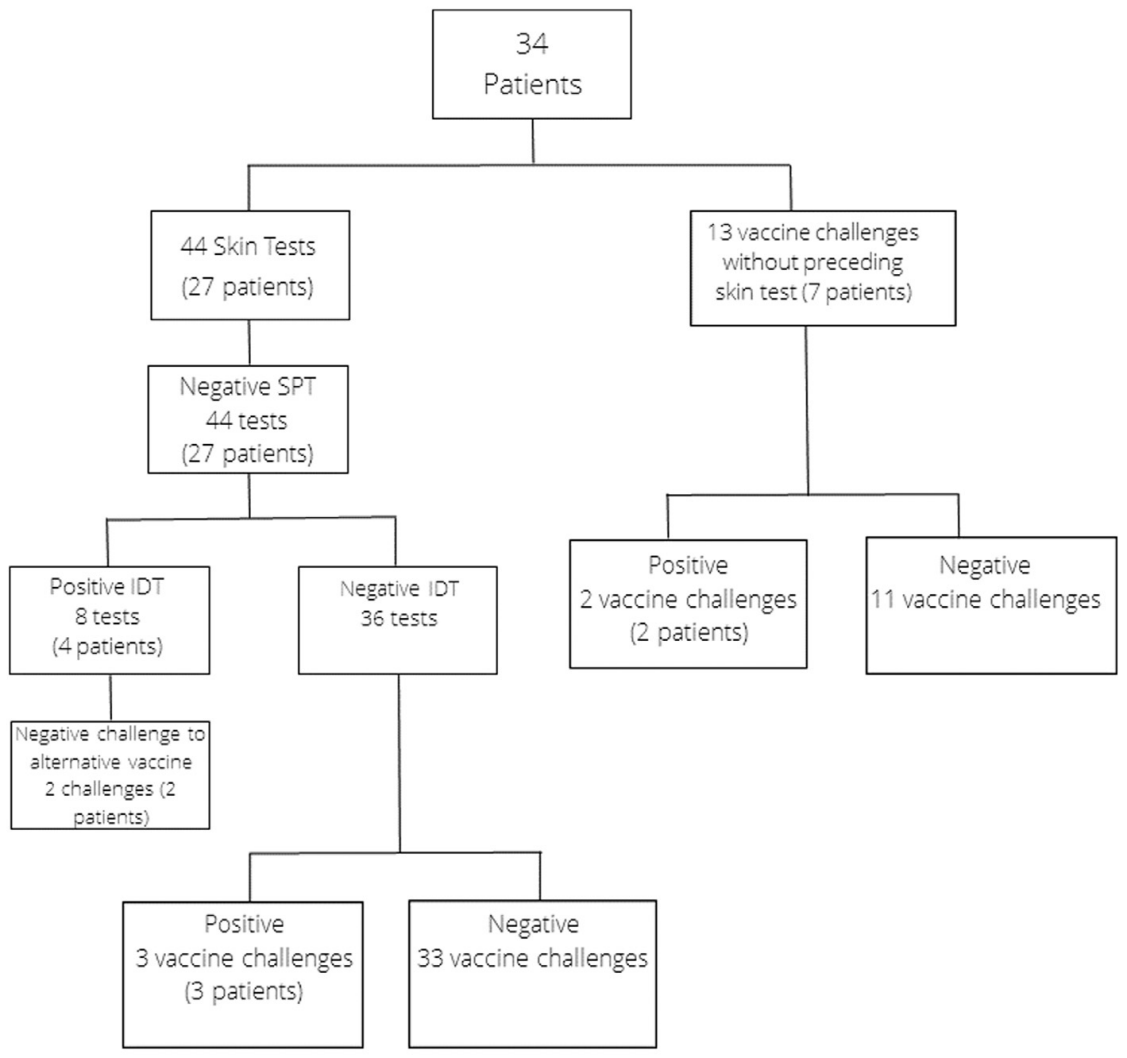
Flowchart of skin test results and vaccine challenges.

Of the 34 patients with a history of allergy to a vaccine or vaccine component, 9 had a confirmed diagnosis of allergy to a vaccine (4 with a positive IDT result, 3 with a positive challenge results and previous negative IDT result, and 2 with positive challenge results without previous IDT) (Fig 3); 32 could be safely vaccinated. Vaccine challenge without a preceding allergy test was performed for 13 vaccines, and skin tests were performed for 44 vaccines (27 patients). Of these 27 patients, 23 tested negative for all antigens investigated (36 tests) and were vaccinated without serious adverse events. One patient tested positive for dT vaccine but negative for diphtheria, tetanus and acellular pertussis (dTPa) vaccine and could be safely vaccinated with the latter. One patient tested positive for influenza vaccine (with thimerosal) but could be safely vaccinated with influenza without thimerosal. Only 2 patients could not be vaccinated; the first tested positive for dT vaccine and dTPa vaccine, and the second tested positive for dT vaccine, dTpa vaccine, influenza vaccine, and influenza vaccine without thimerosal. The result of challenge testing was positive for 4 patients (5 vaccines), but all of the reactions were controlled with oral medication (Table II).

**Table II:** Description of positive challenge testing results

Patient No.	Index vaccine or component	Skin test	Challenge vaccine	Challenge outcome	Medication
6	Gelatin	Not performed	Yellow fever	Pruritus, no rash	Dexchlorpheniramine, 2 mg
7	dT/hepatitis B	Negative result	Hepatitis B	Pruritus after 3 hours + rash, micronodular after 24 h	Prednisone, 40 mg + dexchlorpheniramine, 2 mg
21	Influenza/egg	Negative result	Yellow fever	Pruritus after 25 min, no rash	Hydrocortisone, 100 mg + Fexofenadine, 180 mg
29	Egg	Not performed	Influenza	Pruritus after 15 min, no rash	Fexofenadine, 180 mg
		Negative result	Yellow fever	Pruritus after 20 min, no rash	Fexofenadine, 180 mg

## Discussion

Here, we have described a 7-year period of observation of skin testing and/or challenge performed in adults aged 18 years or older with potential IgE-mediated hypersensitivity reactions related to a vaccine or vaccine component. The number of patients included in the observation period was low, and the time elapsed between the allergy event and first evaluation at a CRIE unit was very long. In part, this can be explained by the lack of patient and physician awareness of the importance of having the vaccination schedule up to date for adults and by the fact that patients are referred only when there is an imminent necessity of vaccination, such as in cases of outbreaks or vaccination requirements for work or travel.

In this study, we observed that the vaccine most frequently associated with a history of allergy was the dT vaccine. Of 9 patients with history of allergy to the DTP or dT vaccine, 2 (22%) had a positive result of cutaneous allergy testing to both the dT and dTpa vaccines, confirming IgE-mediated mechanisms, and they could not be vaccinated. For these patients, we provided a written medical report guiding the use of human antitetanus immunoglobulin in the event of an accident with tetanus risk. The estimates of the incidence of true allergic reactions, or immediate hypersensitivity, in response to the DTP vaccine are higher (1 per 50,000 doses) than for most other vaccines (1 per 500,000-1,000,000 doses^13^) and corroborate our observation.

The vaccine component most frequently associated with allergy was egg, but all of the patients could be vaccinated without serious allergic reaction. Egg allergy is the second most frequent food allergy in children,^14^ and severe egg allergy is classically considered a contraindication to vaccines that use eggs in the vaccine (eg, yellow fever, influenza) production process. In the past few years, however, many studies have reported that inactivated influenza vaccine with less than 1 μg of ovalbumin per dose is safe for recipients with egg allergy, including those with a history of anaphylaxis in response to egg.^15^ It is essential to note that in Brazil, not all available inactivated influenza vaccines indicate the concentration of egg protein on the package inserts and should not be used interchangeably. Yellow fever vaccines can have a higher ovalbumin content depending on the manufacturer (range 0.13-4.42 μg/mL).^15^^,^^16^ However, clinical experience in endemic areas suggests that the vaccine might be safe with adoption of desensitization protocols guided by an allergist.^16^^,^^17^

IDT is the most sensitive skin testing for diagnosis of immediate hypersensitivity; however, there is no consensus on the methodology and interpretation of IDT. We followed the recommendation of Brazilian Association of Allergy and Immunology regarding performing the tests, which is in accordance with the guideline of Societe Francaise d’Anesthesie et Reanimation.^18^ If we were to apply the guideline of the European Academy of Allergy and Clinical Immunology, which considers a test result to be positive when the mean diameter of the wheal after 20 minutes is 3 mm larger than the initial papule (W_20_ ≥ Wi + 3 mm), our results would be the same. Although the results of IDT are more reproducible than those of prick and puncture testing, the possibility of false-positive reactions due to inherent vaccine irritation must be kept in mind,^19^ and as this type of testing carries a higher risk of inducing a systemic allergic reaction, it is imperative that the testing be done in a safe environment with resources for immediate treatment of anaphylaxis.

Our results confirmed those of previous studies showing that overdiagnosis of vaccine allergy is common.^7^ We found that 94.1% of the patients referred to our center could be safely vaccinated; 7 of 34 patients (20.6%) were vaccinated on the basis of clinical history alone, 23 patients (67.6%) were vaccinated after negative skin allergy test results, 2 patients (5.9%) were vaccinated after formulation exchange, and only 2 patients (5.9%) could not be vaccinated. It is important to emphasize that after immunization, some patients can have immediate adverse events that mimic symptoms and signs of hypersensitivity but are not actually allergic reactions to the vaccine administered. Many such reactions can be characterized as immunization stress–related responses.^20^^,^^21^

As stated by Kelso, in the past 10 years great progress has been made in the understanding of and approach to patients with previous reactions to vaccine constituents or vaccine administration, and nowadays many patients can be safely vaccinated without additional tests.^20^ Adoption of a systematic approach by an experienced team and, when indicated, with skin testing can reduce the number of contraindications and missed opportunities for vaccination. Unlike laboratory tests that measure specific serum IgE levels, skin tests are simple, low-cost, point-of-care procedures that could easily be adopted even in low- and middle-income countries. The possibility of verifying the presence of a hypersensitivity reaction is critical to avoiding the use of medical exemptions to circumvent mandatory vaccination, particularly by those in the growing antivaccine movements.^22^

In conclusion, overdiagnosis of vaccine allergy is common and is considered a public health problem. Implementing a multidisciplinary team (allergist and infectious disease specialist) in CRIE units allows the adoption of a systematic approach of patients with history of vaccine allergy for safe immunization, contributing to reduction of unnecessary vaccine schedule interruption.
